# Satisfaction with the life of Polish women experiencing domestic violence

**DOI:** 10.3389/fpubh.2023.1073612

**Published:** 2023-02-13

**Authors:** Agnieszka Konstancja Pawłowska-Muc, Anna Bogusława Pilewska-Kozak, Grażyna Stadnicka, Jan Jakub Kęsik, Beata Dobrowolska

**Affiliations:** ^1^Independent Public Complex of Healthcare Facilities, Pionki, Poland; ^2^Chair of Obstetrics and Gynaecology, Department of Obstetrics and Gynaecology Nursing, Faculty of Health Sciences, Medical University of Lublin, Lublin, Poland; ^3^Chair and Department of Vascular Surgery and Angiology, Medical University of Lublin, Lublin, Poland; ^4^Chair of Integrated Nursing Care, Department of Holistic Care and Nursing Management, Faculty of Nursing, Medical University of Lublin, Lublin, Poland

**Keywords:** domestic violence, women, wellbeing, satisfaction with life, relations in the family

## Abstract

**Introduction:**

Domestic violence against a woman, inflicted by her husband/partner, disrupts the socially recognized model of partnership and family life and endangers the health and life of the victim. The aim of the study was to assess the level of satisfaction with the life of Polish women experiencing domestic violence and compare it to results of women not experiencing domestic violence.

**Methods:**

A cross-sectional study was conducted on a convenience sample of 610 Polish women divided in two groups: victims of domestic violence (Group 1, *n* = 305) and women not experiencing domestic violence (Group 2, *n* = 305).

**Results and Conclusions:**

Most Polish women experiencing domestic violence are characterized by low life satisfaction. The mean value of life satisfaction in Group 1 was 13.78, SD = 4.88, significantly lower when compared to Group 2 (M = 21.04, SD = 5.61). Their satisfaction with life is related, among other things, to the form of violence inflicted upon them by their husband/partner. Abused women with low life satisfaction are most often victims of psychological violence. The most common cause is the perpetrator's addiction to alcohol and/or drugs. Assessment of their life satisfaction is unrelated to help-seeking and to the occurrence of violence in their family home in the past.

## Introduction

Domestic violence is these days an increasingly visible and worryingly growing phenomenon (1–3). It manifests as physical violence, psychological abuse, neglect, threats and acts of violence committed by the perpetrator against the victim, which take place in the household. Domestic violence can be inflicted by a woman or by a man against their spouse/life partner. Forms of physical violence include hitting, slapping, kicking, choking, pushing, throwing objects and other acts that cause injuries and threaten the victim's safety. Victims who experience psychological violence, such as verbal abuse in the form of harsh words, are suspicious and feel too restricted in their lives (4). WHO reports that one in three women worldwide have experienced physical and sexual violence from their partners (5).

Domestic violence against a woman, inflicted by her husband/partner, disrupts the socially recognized model of partnership and family life and endangers the health and life of the victim. Various health and mental health problems constitute a significant issue. According to a WHO report of 2013, the health consequences of violence are divided into physical, mental, sexual and those that end in death (6, 7). Other authors divide them into physical, health and psychological consequences (8). Yet another division distinguishes between medical, psychological and social consequences (9). Negative effects include physical consequences, which are divided into immediate and distant. As for the immediate consequences, their symptoms occur immediately or within a short time after the violence was inflicted, while distant ones occur after a certain period of time (10, 11). Numerous authors from different professions and disciplines have portrayed this problem in quite a few easily accessible scientific publications (10–15). The most dramatic consequence of domestic violence is the murder of a woman committed by her husband/partner (11, 12, 15, 16).

This issue has been of interest to people from various professions and scientific disciplines for many years. New publications on the subject are constantly appearing, but the issue is still considered poorly understood. In the available literature, the phenomenon of violence, not solely domestic violence, is often showed as a widespread phenomenon, which is present in many countries. Given the various consequences of domestic violence, life satisfaction in women who experiencing it is reduced (17–23). A study by Hui and Constantino (24) has proven that women experiencing intimate partner violence have lower self-esteem, are more likely to have a depressed mood, need psychological support and have poorer life satisfaction.

In Poland the issue of domestic violence and how it affects women who experience it has been a subject of extensive research (25–28), however to the best of our knowledge, the life satisfaction of this group of women and comparative analyses in relation to women who do not experience violence are not the subject of any published research findings. By undertaking the research, we intended for the findings to serve as a point of reference in the search for new solutions, especially in the field of support for women who are victims of domestic violence, and actions strengthening their mental wellbeing.

### Aim of the study

The aim of the study was to assess the level of satisfaction with the life of Polish women experiencing domestic violence and compare it to results of women not experiencing domestic violence.

## Materials and methods

### Study design

A cross-sectional study was conducted according to the STROBE (Strengthening the Reporting of Observational studies in Epidemiology) guidelines (29) on two groups of women: a group of victims of domestic violence (Group 1-Gr1) and a group of women who have not experienced violence (Group 2-Gr2), in five provinces of Poland.

### Participants

A convenience sample of 610 women, including 305 victims of domestic violence (Gr1) and 305 women who had not experienced violence (Gr2). Women from Gr1 were investigated in thirteen different help centers for victims of domestic abuse located in five Polish provinces: Mazowieckie, Podkarpackie, Swietokrzyskie, Lubuskie, Podlaskie (out of 16 provinces in Poland). A total of sixteen help centers in Poland were approached. Finally, the consent was obtained from the Heads of the thirteen above-mentioned centers. These were the centers that expressed their willingness to cooperate with researchers.

Help centers are institutions which provide specialized assistance to women experiencing various forms of violence. They offer various form of assistance, such as psychotherapy, occupational therapy and psychological counseling. The centers employ a team of specialists consisting of a psychologist, a clergyman (priest), an occupational therapist, a social worker and a lawyer.

The inclusion criteria for women who constituted Gr1 included:

1. Age over 18 years.
2. Experience of domestic violence (one or more types of violence) inflicted by husband/partner.
3. Use of institutional care.
4. Being in good health at the time of the study.
5. Giving written informed consent for participation in the study.

The convenience sample of women in Gr2 was recruited from patients of outpatient obstetric-gynecologic clinics in central Poland (the city of Radom). The inclusion criteria for women in Gr2 were the same as for Gr1, with the exception of point 2 and 3. Prior to the study, each candidate for Gr2 was asked to complete a self-diagnostic questionnaire, entitled: “Are you experiencing domestic violence?”. This questionnaire, developed by The Polish Nationwide Emergency Service for Victims of Domestic Violence “Blue Line”, consisted of 28 questions, with only two possible answers for each-yes or no.^1^ The questionnaire addressed, among other things, the following questions: Does your partner treat you in a way that hurts you? Does your partner throw insults at you, call you names? Does your partner force you to do things that demean you? Does your partner tell you that they know what is right for you and you don't? Does your partner ridicule or insult people you like and value? Do you feel uncomfortable when it comes to meeting your friends in the presence of your partner? Does your partner constantly criticize you? Has your partner told you that you are stupid, that you are not good for anything? Does your partner not allow you to see or talk to friends, family? Has your partner ever pushed you, pulled you or slapped you? Have you ever been so beaten by your partner that you required medical attention or hospital treatment? Does your partner blame you for his violent behavior, telling you that you are to blame for everything? Has your partner forced you to have sexual intercourse? Has your partner threatened to beat up your friends if they try to help you? Does your partner take your money, make you ask for it or simply refuse to give it to you? Does your partner be have toward your children in a way you find objectionable? Do you often get depressed? Do you have suicidal thoughts? Even one positive answer excluded the candidate from participation in the study, because, according to the authors of the questionnaire, it was indicative of a high probability of experiencing violence. Such candidate was therefore not eligible for Gr2.

### Measurements

The study relied on a questionnaire which consisted of two parts:

The first part, a self-designed questionnaire, consisted of 37 questions, addressed to Gr1, and 32 questions addressed to Gr2. Its aim was to collect information on the socio-demographic data of the participants (age, place of residence, marital status, level of education and occupational activity, socio-economic status, having offspring), subjective assessment of health and current diseases, attitude to preventive medical check-ups and selected elements of lifestyle (habits, addictions, physical activity and leisure time activities). The questionnaire for Gr1 also included questions on the domestic violence the respondent was experiencing, including its forms, causes and types. In constructing this part of the questionnaire, the literature on the subject (25, 26, 30–35) and suggestions and comments from experts in the field of gynecology, psychology and psychiatry, were used.

The second part included the Satisfaction With Life Scale (SWLS) by Diener et al. (36) adapted by Juczyński (37), designed for individual and group examination of adults, both healthy and ill. It consists of five statements. The respondent assesses the extent to which each statement relates to their life experience. The questionnaire uses a typical Likert scale, with seven possible answers and the following scoring: 1-strongly disagree, 2-disagree, 3-slightly disagree, 4-neither agree nor disagree, 5-slightly agree, 6-agree, 7-strongly agree. For each respondent, the scores are then aggregated. They range from 5 to 35 points. The aggregated scores are then converted into sten units, interpreted in the following way: 7–10 sten means high life satisfaction, 5–6 sten means average life satisfaction, and 1–4 sten is interpreted as low life satisfaction. The result of the measurement is an overall index of life satisfaction—the higher the score, the greater the feeling of satisfaction (37).

Life satisfaction is an overall assessment of general feelings and attitudes about one's life at a particular point in time, ranging from negative to positive. Both positive and negative experiences relate to cognitive-evaluative well-being. It is defined as the cognitive appraisal of individuals who reflect on their lives as a whole or on certain areas of their lives, such as relationships, the environment or the self (36). Individual life satisfaction can be measured in relation to the past life, and present or expected life satisfaction. One might be tempted to say that life satisfaction is a person's cognitive and reflective appraisal or evaluation of how well and how fulfilled they are in life. The Satisfaction With Life Scale (SWLS) used in this study is supposed to assess a person's satisfaction with their life as a whole (38). It is a tool for measuring subjective well-being, in terms of cognitive and emotional aspect. Such understanding of the concept of 'life satisfaction' has been adopted in this study. The Scale does not assess satisfaction with various areas of life, such as health, family, marriage and living conditions.

The stage of preparing the research tools was completed with a pilot study conducted over a period of two months. The pilot study involved thirty women who met the inclusion criteria for Gr 1 and Gr2. Women who participated in the pilot study did not provide any comments to the questionnaire, nevertheless it was decided that the results of the pilot study were ultimately excluded from this study.

### Data collection

In the help centers where the study was carried out each participant was informed of the aim of the research before filling out the questionnaire. It was also explained how to complete the questionnaire and the participants were ensured that the results would remain anonymous. The participants filled out the questionnaires on their own, with no time limit imposed. The meetings were held in separate rooms. All women were granted privacy, peaceful and quiet atmosphere and support. They were offered an opportunity to have a relaxed conversation before filling out the questionnaire, could ask questions when completing the task, and share their observations, doubts and anxieties.

It happened many times that filling out the questionnaire was accompanied by emotions, which were the result the wrongs suffered in the past. In such situations, the interviewers and questionnaire collectors (nurses instructed in advance to do perform this task) heard them out, provided support, and encouraged them to maintain frequent contact with the psychologist employed by the help center.

A total of 660 women took part in the study. 20 women did not consent to participate (15 from Gr1 and 5 from Gr2). Most of them did not give any reason for their refusal or stated that they were doing so for personal reasons, which they did not explain further. In addition, thirty questionnaires were incomplete and they were excluded from this study.

### Ethical issues

The study was conducted in accordance with the protocol approved by the Bioethics Committee of the Medical University of Lublin (No. KE-0254/299/2015) and in accordance with the tenets of the Declaration of Helsinki.

Each participant was informed of the aim of the research before filling out the questionnaire. It was also explained how to complete the questionnaire and the participants were ensured that the results would remain anonymous. The participants filled out the questionnaires on their own, with no time limit imposed.

### Data analysis

The collected study material was analyzed using the statistical package IBM SPSS Statistics (v. 25). Quantitative variables were described by mean, standard deviation, median, lower quartile, upper quartile, as well as minimum and maximum values. As for qualitative variables, the abundance and percentage of the indicated categories were given.

Assuming differences between Gr1 and Gr2, appropriate statistical procedures were applied. Firstly, the normality of distribution of the analyzed variables was checked (Shapiro-Wilk test), and then the other assumptions of parametric tests (homogeneity of variance—Levene's test, and the equality of proportions of the compared groups—chi-square test of concordance). Comparisons were made using the Student's t-test for independent groups. The test was used to verify the hypothesis about the equal value of the means of the studied variable in the two populations. To determine the relationship between the variables, measured on a qualitative scale, a chi-square test was used. It was used when the dependent variable was already measured on a nominal scale. The basis of the testing procedure was the comparison of two types of counts, located in the cells of a cross-tabulation containing the variables considered in the analysis. These were the observed abundances (the actual, empirical number of observations), and the expected abundances (the hypothetical abundances we expect to see in the table cells, assuming the variables are independent). The obtained results were assumed to be statistically significant at *p* < 0.05. The results are presented to the nearest thousandths - for values less than 0.001, the notation *p* < 0.001 was used.

## Results

### Characteristic of study participants

One in two (310; 50.8%) participants completed tertiary education, one in three (210; 34.4%) completed secondary education, and only 90 (14.8%) did not attend a secondary school of any type. The differences between the groups were statistically significant, as women with a higher level of education dominated Gr2 (*p* < 0.001). More than half (416; 68.2%) of the women held permanent employment, and one in three (194; 31.8%) was not in permanent employment. The differences between the groups proved statistically significant in favor of Gr2 (*p* < 0.001). At the time of the study the majority of women were living in their own flats (‘own' understood in a colloquial way) (466; 76.4%). Others stayed in their parents' flat (84; 13.8%), in a shelter or a single mother's home (32; 10.5%) or in rented accommodation (28; 4.6%). The differences between the groups proved statistically significant in favor of Gr2 (*p* < 0.001). The majority of the women were in a civil partnership or marriage (468; 78.8%), and the rest of them (142; 21.2%) were not in a relationship at the time of the study. The differences between the groups proved statistically significant in favor of Gr2 (*p* < 0.027) (Table 1).

**Table 1:** Sociodemographic data of the participants.

**Age**	**Gr1 (21–75 yrs)**			**Gr2 (19–70 yrs)**			**Significance**
	**Me**	**Q1**	**Q2**	**Me**	**Q1**	**Q2**	***p*** = **0.323**
	42	35	50	45	32	50	
**Place of residence**	***n***	**%**		***n***	**%**		
City 401 (65.7%)	197	64.6		204	66.9		*p* = 0.609
Village 209 (34.3%)	108	35.4		101	33.1		
**Education**							
Less than secondary primary, junior high, vocational 90 (14.8%)	88	28.9		2	0.7		*p* < 0.001
Secondary 210 (34.4%)	104	34.1		106	34.7		
Above secondary tertiary: Bachelor's degree, Master's degree 310 (50.8%)	113	37.0		197	64.6		
**Permanent employment**							
Yes 416 (68.2%)	148	48.5		268	87.9		*p* < 0.001
No 194 (31.8%)	157	51.5		37	12.1		
**Current accommodation**							
In own flat 466 (76.4%)	224	73.4		242	79.3		*p* < 0.001
In parents' flat 84 (13.8%)	37	12.1		47	15.4		
In a shelter or single mother's home 32 (5.2%)	32	10.5		0	0.0		
In a rented flat 28 (4.6%)	12	3.9		16	5.2		
**Marriage or civil partnership**							
Yes 468 (78.8%)	221	72.5		247	81.0		*p* = 0.027
No 142 (21.2 %)	84	27.5		58	19.0		

Q1, lower quartile; Me, median; Q3, upper quartile.

### Experiencing domestic violence

The presence of violence in the past, in the family home of the participant, was reported by one in five (119; 19.5%) women, while the majority (491; 80.5%) denied it. The differences between the groups proved to be statistically significant, as women in Gr1 reported this fact more often than those in Gr2 (*p* < 0.001) (Table 2).

**Table 2:** Violence experienced in the past—in the family home of participants.

**Violence in the family home**	**Gr1**		**Gr2**		**Significance**
	***n***	**%**	***n***	**%**	
Yes 119 (19.5%)	93	30.5	26	8.5	*p* < 0.001
No 491 (80.5%)	212	69.5	279	91.5	

The women in Gr1 were asked about the causes of domestic violence which they are experiencing currently in their homes, the forms of violence, about the perpetrator, and whether they sought help. Detailed data on this subject are presented in Table 3.

**Table 3:** Causes, forms, and perpetrators of domestic violence experienced by participants in Gr1.

	***n***	**%**
**Cause of domestic violence** ^*^		
Addictions of the husband/partner to alcohol and/or drugs	212	69.5
Jealousy, lack of trust in the relationship	116	38.0
Differences of opinion	89	29.2
Household chores	85	27.9
Financial difficulties	81	26.6
Incompatibility of characters	69	22.6
Differences of opinion on raising children	63	20.7
Difficult to say	39	12.8
Loss of job	29	9.5
Failures at work	26	8.5
Total^*^	881	100.0
**Forms of violence** ^*^		
Psychological	282	92.5
Physical	218	71.5
Neglect	96	31.5
Economic	84	27.5
Sexual	61	20.0
Total^*^	741	100.0
**Perpetrator**		
Husband	264	86.6
Partner	41	13.4
Total	305	100.0
**Seeking help**		
Yes	140	45.9
No	165	54.1
Total	305	100.0

* Participants could provide more than one answer.

### Satisfaction with the life of women with and without experience of domestic violence

Women from Gr2 presented a significantly higher satisfaction with life (*p* < 0.001) (Table 4).

**Table 4:** Satisfaction With Life (SWLS) of women in Gr1 and Gr2.

**Satisfaction With Life (SWLS) in women**	**Gr1**		**Gr2**		**Significance**
**Statements**	**M**	**SD**	**M**	**SD**	
In most ways my life is close to my ideal	2.33	1.21	3.84	1.32	*p* < 0.001
The conditions of my life are excellent	2.66	1.21	4.00	1.33	
I am satisfied with my life	3.11	1.38	4.81	1.22	
So far I have gotten the important things I want in life	3.21	1.33	4.42	1.43	
If I could live my life over, I would change almost nothing	2.47	1.37	3.96	1.68	
**SWLS scores**					*p* < 0.001
General score	13.78	4.88	21.04	5.61	
Sten score	3.19	1.68	5.71	2.00	

M, mean; SD, standard deviation.

Life satisfaction was low in every second woman (314; 51.5%), average in every third (181; 29.7%) and only in 115 (18.9%) women the score was high. The differences between the groups proved to be statistically significant to the disadvantage of Gr1, as it was characterized by a majority of low scores (*p* < 0.001) (Table 5).

**Table 5:** Scores for satisfaction with life (SWLS) after conversion to sten scores.

**Scores**	**Gr1**		**Gr2**		**Significance**
	***n***	**%**	***n***	**%**	
Low (1–4 sten) 314 (51.5%)	235	77.0	79	25.9	*p* < 0.001
Average (5–6 sten) 181 (29.7%)	61	20.0	120	39.3	
High (7–10 sten) 115 (18.9%)	9	3.0	106	34.8	

Life satisfaction scores appeared to be significantly related to the form of violence experienced, as women with low and average life satisfaction dominated in the group experiencing psychological violence (*p* = 0.019). As for the other studied characteristics, there was no such relationship (*p* > 0.05) (Table 6).

**Table 6:** Satisfaction with life vs. study participants characteristics.

**Studied characteristics**	**Low scores 235 (77.0%)**		**Average scores 61 (20.0%)**		**High scores 9 (3.0%)**	
	***n***	**%**	***n***	**%**	***n***	**%**
**Form of domestic violence**						
Psychological 282 (92.5%)	217	37.0	58	43.6	7	33.3
Physical 218 (71.5%)	171	29.1	42	31.6	5	23.8
Neglect 96 (31.5%)	75	12.8	16	12.0	5	23.8
Economic 48 (27.5%)	68	11.6	12	9.0	4	19.9
Sexual 61 (20.0%)	56	9.5	5	3.8	0	0.0
Significance	*p* = 0.019					
**Help seeking**						
No 165 (54.1%)	122	51.9	35	57.4	8	88.9
Yes 140 (45.9%)	113	48.1	26	42.6	1	11.1
Significance	*p* = 0.078					
**Violence in the family home**						
No 212 (69.5%)	163	69.4	44	72.1	5	55.6
Yes 93 (30.5%)	72	30.6	17	27.9	4	44.4
Significance	*p* = 0.598					

## Discussion

### Profile of women in terms of selected sociodemographic data

Age did not significantly differentiate the studied groups (*p* < 0.05). In Gr1 it ranged from 21 to 75 years of age, and in Gr2 from 19 to 70 years. These results proved to be different from those found in the literature (39, 40), because women who participated in this study were much younger.

As for the place of residence of the participants, there were no significant differences between the groups - one in three resided in the countryside and the others in the city. A similar distribution of data on this topic (27.92 and 72.08 and 38.0 and 62.0%) was presented by other authors (27, 28). On this basis, however, it could not be inferred that domestic violence is experienced more often by urban than rural women. Statistically fewer women live in rural areas than in urban areas, hence the participation of rural women in various studies might have been lower. It may also be that women in urban areas, feeling more anonymous, were more likely to disclose their problem, as suggested by some participants (27, 28, 41, 42).

Women in Gr1 had a significantly lower level of education than the participants in Gr2 (*p* < 0.001). In Gr1 there was a significantly higher proportion of women with less than secondary education, and in Gr2 more participants had above secondary education. It is worth noting, however, that in both groups the numerical distribution of the data was similar, with most women having above secondary education, followed by those with secondary and less than secondary education. Reports from the literature presented different distribution. A nationwide study, commissioned by the Ministry of Labor and Social Policy in 2014, shows that among victims of domestic violence the largest group was composed of women with secondary education (39.7%), followed by those with tertiary education (34.0%), and less than secondary education (26,3%) (27). In another study, conducted three years later for the same commissioner, domestic violence was most common among women with less than secondary education (41.07%), followed by women with secondary (32.15%) and tertiary education (26.79%) (28). A similar distribution of the level of education in the studied groups was observed by other authors (43). So, all groups studied by different investigators comprised women with different levels of education - from primary to tertiary.

At the time of the study, the majority of women (68.2%) held permanent employment, with nearly twice as many permanently employed participants in Gr2 than in Gr1. Half of the participants in Gr1 (51.5%) were unemployed. Different results were presented in the report entitled ?Nationwide diagnosis of support infrastructure for persons experiencing violence and evaluation of the effectiveness and efficiency of the forms of assistance granted” (28). According to that study, 42.55% of abused women held permanent employment, 4.26% were self-employed, 41.13% were unemployed, 7.80% were retired, 4.26% were on a disability pension, and 2.84% were still gaining education (28). The distribution was, however, different in the report from earlier studies, where the largest group of abused women embraced participants who were working or studying (52.5%). 41.9% of participants were unemployed, and 5.6% were on a disability pension (27). In yet another study a surprisingly small percentage of studied women did not have a job (16.4%) (41).

When analyzing the reasons for not being in permanent employment, which were reported by the participants, it was noted that being on a disability pension and losing a job were the reasons quoted only by victims of violence. Whether and to what extent this was related to the experience of domestic violence and its health consequences cannot be determined on the basis of the collected material. This is because it is not known on what grounds the certification of incapacity for work was issued by the evaluation board. Also the cause of the chronic illness they claimed to have was not known to the investigators, as well as how long it had lasted and how often the woman was on sick leave. This may have contributed to the loss of job. However, clarifying these issues requires separate research.

At the time of the study, the majority of participants were married, with a slightly fewer number in Gr1 (60.7%) than in Gr2 (67.5%). However, the group comprising divorced participants was more than three times as big. The cause of the marital breakdown is not known, as such data were not collected in this study. A similar distribution of data regarding the marital status of women experiencing domestic violence was observed by another author (41). However, the proportion of divorced participants was lower (13.2%).

Another interesting fact was that participants in the study group remained in a marital/partnership relation. This characteristic significantly differentiated Gr1, in favor of Gr2. The duration of their relationships, on the other hand, was irrelevant in this regard, and amounted to an average of 18.48 ± 10.95 years. A slightly shorter, although also long, duration of the relationship [16 ± 9.85 years on average] was observed by other authors (39).

### Satisfaction with the life of Polish women experiencing domestic violence

In the material presented in this study, low life satisfaction was observed almost three times more frequently in Gr1 than in Gr2. The opposite was true for average and high life satisfaction. The differences between these groups proved statistically significant, to the disadvantage of the participants in the Gr1.

As a result the investigators decided to check if there was a relationship between the assessment of life satisfaction in Gr1 and the form of domestic violence women experienced, their help-seeking, and the occurrence of violence in the past—in their family home. Such an association was detected only with regard to the form of violence experienced. The other two characteristics were insignificant in this regard. In reviewing the literature on life satisfaction among abused and non-abused women, only two papers contained the results of comparative studies similar to those presented in this study (19, 22). The results these researchers obtained regarding life satisfaction were similar to those obtained in this study.

The reports in the available literature (17, 18, 20, 21) prove that life satisfaction was studied only among women experiencing domestic violence, and the aim of the researchers was to look for its determinants. There was no comparison with women not experiencing violence.

A regression analysis, compiled by Hassan and Malik (21) showed that domestic violence significantly affects the so-called psychological well-being of women, in particular their life satisfaction, self-esteem and incidence of depression. Furthermore, these researchers found a significant negative correlation between domestic violence and certain—what they termed—indicators of psychological well-being, i.e. life satisfaction, self-esteem and levels of dispositional optimism. Translating this to the subject of their study, it should be understood that the persistence or escalation of domestic violence against women lowered the aforementioned indicators.

The reasons for the lowered satisfaction with life in victims of domestic violence were supposed to be related to their living situation. The main reason was the increased exposure to stress (17, 20, 21), but there were also certain socio-demographic characteristics that affected the level of life satisfaction (18). In the study material by Abbas and Shah (17) a significant negative relationship was detected between the perceived stress and life satisfaction of abused women, and the perceived stress and their self-esteem. These observations were consistent with reports by other authors (21). Other observers noted a favorable change in life satisfaction scores in women after 13 sessions of individual psychotherapy (20). In their report the improvement in the scores was the result of a reduction of symptoms of anxiety, stress, depression and post-traumatic stress disorder (PTSD) in the studied women. Psychological help in cases of violence is of particular importance. Unfortunately, our research has shown that more than half of Gr1 women experiencing violence did not seek help, not just psychological. In addition, our research shows that women from Gr1 statistically more often than women from Gr2 experienced violence in the past, in their family homes. Also for this reason, long-term psychological help would benefit abused women, changing some patterns of reactions in situation of violence.

There is also an interesting study that demonstrates a relationship between the evaluation of life satisfaction in victims of domestic violence and their selected sociodemographic characteristics (18). Lower life satisfaction was most often observed among rural residents, among women with a higher level of education, and among those working in the private sector. Similar low level of life satisfaction was also reported by the unemployed (44). The reasons, therefore, for low life satisfaction in victims of domestic violence can be varied, complex and not always just related to the abuse. In the literature, this issue is quite rarely addressed (45).

Research related to domestic violence against women is increasingly concerned with assessing their quality of life, rather than life satisfaction (46–51). Therefore, the subject of our study encourages further inquiry.

### Limitations of the study

Experiencing of domestic violence is a difficult situation for the abused woman and those around her. Conducting research with this group is not easy. Despite assurances of anonymity of the information obtained from them, they are generally distrustful, suspicious and reluctant to agree to reveal their experiences, feelings and behaviors. The participants may have felt embarrassed to provide honest answers when filling out the questionnaire about their experiences. A limitation of the research is certainly its nature - a cross-sectional study that makes it impossible to infer a cause-and-effect relationship. Another limitation refers to the participants' self-assessment regarding the issue under investigation.

The obtained results showed that quantitative surveys on sensitive data, which undeniably include information on the experience of violence, do not fully allow for a thorough description of the problem. Perhaps it could be interesting to do a qualitative study in the future, which would show a picture of the abused woman in terms of her life satisfaction.

## Conclusion

Most Polish women experiencing domestic violence are characterized by low life satisfaction. Their satisfaction with life is related, among other things, to the form of violence inflicted upon them by their husband/partner. The abused women with low life satisfaction are most often victims of psychological violence. The most common cause is the perpetrator's addiction to alcohol and/or drugs. Assessment of their life satisfaction is unrelated to help-seeking and to the occurrence of violence in their family home.

It is important for professionals in various fields, not only medical professionals, to learn about the problems abused women face, including their life satisfaction. This would allow them to better understand some of the attitudes and behaviors of the victims. It would also facilitate communication, which is very difficult for various reasons, when working directly with the abused is required. What is more—any activities can be useful in finding new solutions aimed at improving the quality of care provided to women experiencing domestic violence, not only for diagnosis and treatment, but above all for prevention and care. Learning about the phenomenon of violence in the context of life satisfaction of abused women will also make it possible to revise the training curricula of medical personnel to whom women with various health problems are referred. Curricula should take into account the aspects highlighted by the results of this study, specifically that abused women in a majority do not seek the help and that in the past they often experienced violence in their family home.

## Data availability statement

The original contributions presented in the study are included in the article/supplementary material, further inquiries can be directed to the corresponding authors.

## Ethics statement

The studies involving human participants were reviewed and approved by Medical University of Lublin. The patients/participants provided their written informed consent to participate in this study.

## Author contributions

AP-K, AP-M, GS, and BD: conceptualization and drafting—revising and editing. AP-K, AP-M, and GS: methodology. BD: formal analysis and supervision. AP-M: research. AP-M, AP-K, GS, JK, and BD: data curation. All authors have read and accepted the published version of the manuscript.

## Footnotes

^1^ https://www.niebieskalinia.pl/aktualnosci/archiwum/kwestionariusz-dla-kobiet-czy-doznajesz-przemocy-w-rodzinie

## References

[1] Helios J Jedlecka W. Współczesne Oblicza Przemocy. Wrocław: Zagadnienia wybrane Uniwersytet Wrocławski (2017).

[2] Report EIGE. European Institute for Gender Equality-EIGE in Brief. Luxembourg: Publications Office of the European Union. (2017).

[3] Raport GREVIO dotyczacy rozwiazań prawnych i innych słuzacych wdrozeniu postanowień Konwencji Rady Europy o zapobieganiu i zwalczaniu przemocy wobec kobiet i przemocy domowej (konwencja stambulska) – ocena wyjściowa POLSKA z dnia 23 czerwca 2021 r. Available online at: https://bip.brpo.gov.pl/sites/default/files/2022-02/RAPORT_GREVIO_PL.pdf (accessed September 20, 2022).

[4] Afdal A Arnaldi A Nirwana H Alizamar A Zikra Z Ilyas A. Increasing life satisfaction of domestic violence victims through the role of supporting group therapy on social media. Adv Soc Sci Edu Human Res atlantis Press. (2019) 372:139–44. Available online at: file:///C:/Users/beatadobrowolska/Downloads/125925070.pdf (accessed September 20, 2022).

[5] World Health Organization. Violence Against Women: Intimate Partner and Sexual Violence Against Women. (2021). Available online at: www.who.int/reproductivehealth (accessed September 20, 2022).

[6] World Health Organization. Department of Reproductive Health and Research, London School of Hygiene and Tropical Medicine, South African Medical Research Council. Global and Regional Estimates of Violence Against Women: Prevalence and Health Effects of Intimate Partner Violence and Non-Partner Sexual Violence. Geneva: WHO (2013).

[7] World Health Organization. Global and Regional Estimates of Violence Against Women: Prevalence and Health Effects of Intimate Partner Violence and Non-Partner Sexual Violence. Department of Reproductive Health and Research, London School of Hygiene and Tropical Medicine, South African Medical Research Council. Geneva: WHO (2013).

[8] Zboina B Wieczorek I. Przemoc domowa wobec kobiet. Acta scientifica academiae ostroviensis sectio A, nauki humanistyczne. Społeczne i Techniczne. (2013) 1: 319–44.

[9] Leoniuk K Nowakowska H Sobczak K. Zadania pielegniarki w systemie przeciwdziałania przemocy domowej. Probl Pieleg. (2013) 21:397–402.

[10] Kluczyńska S. Problemy zdrowotne i formy pomocy dorosłym ofiarom przemocy domowej. [W:] Danielewicz D, Rola J (red.): Stare dylematy i nowe wyzwania w psychoterapii. Wyd. Warszawa: Akademii Pedagogiki Specjalnej (2017) 103-118.

[11] Łukowska K. Medyczne i psychologiczne skutki przemocy w rodzinie jako problem zdrowia publicznego. Hyg Pub Health. (2018) 53:24–30.

[12] Report FRA. Eurpean Union Agency for Fundamential Rights. Przemoc wobec kobiet: badanie przeprowadzone w skali UE. Wyd. Urzad Publikacji Unii Europejskiej, Luksemburg: (2014) 7-44. Available online at: https://C:/Users/Windows/Downloads/fra-2014-vaw-survey-at-a-glance-oct14_pl%20(6).pdf (accessed September 20, 2022).

[13] Almis BH Kütük EK Gümüştaş F Çelik M. Risk factors for domestic violence in women and predictors of development of mental disorders in these women. Noro Psikiyatr Ars. (2018) 55:67–72. 10.29399/npa.19355 30042644 PMC6045806

[14] Chhabra S. Effects of societal/domestic violence on health of women. J Women's Health Reprod Med. (2018) 2:1–7. Available online at: https://www.imedpub.com/articles/effects-of-societaldomestic-violence-on-health-of-women.pdf (accessed 20 September 2022).

[15] Chisholm CA Bullock L Ferguson JE. Intimate partner violence and pregnancy: epidemiology and impact. Am J Obstet Gynecol. (2017) 217:141–4. 10.1016/j.ajog.2017.05.042 28551446

[16] Flynn S Gask L Appleby L Shaw J.Homicide-suicide and the role of mental disorder: a national consecutive case series. Soc Psychiatry Psychiatr Epidemiol. (2016) 51:877–84. 10.1007/s00127-016-1209-4 27086087 PMC4889623

[17] Abbas H Shah MW. Relationship between perceived stress, life satisfaction and self esteem among females facing domestic violence. ASH. (2017) 3:73–5. 10.11648/j.ash.20170305.15

[18] Jadav SS Suvera PS. Life satisfaction: A Comparative study of the urban and rural areas women victims of domestic violence. J Contemp Psychol Res. (2016) 3:41–52.

[19] Liu M Xue J Zhao N Wang X Jiao D Zhu T. Using social media to explore the consequences of domestic violence on mental health. J Interpers Violence. (2018) 31-21. 10.1177/0886260518757756 29441804

[20] Habigzang LH Schneider JA Frizzo RP Freitas CPP. Evaluación del impacto de unaintervención cognitive—Conductual paramujeres en situación de violenciadoméstica en Brasil. Universitas Psychologica. (2018) 17:1–11. 10.11144/Javeriana.upsy17-3.eicb

[21] Hassan S Malik AA. Psycho-social correlates of intimate partner violence. Pak J Psychol Res. (2012) 27:279–95. Available online at: https://citeseerx.ist.psu.edu/document?repid=rep1&type=pdf&doi=888c8914bf712b8983b509550c07670d4a682fa9 (accessed September 20, 2022).

[22] Sinha A Ram F. Subjective wellbeing among young married women: assessing their satisfaction with life in North Dinajpur District of West Bengal, India. Social Science Spe. (2017) 3:184–94.

[23] Sinha S. Multiple roles of working women and psychological well-being. Ind Psychiatry J. (2017) 26:171–7. 10.4103/ipj.ipj_70_16 30089965 PMC6058447

[24] Hui V Constantino RE. The association between life satisfaction, emotional support, and perceived health among who experienced intimate Partner violence (IPV)−2007 behavioral risk factor surveillance system. BMC Public Health. (2021) 21:641. 10.1186/s12889-021-10665-4 33794819 PMC8015742

[25] Przeperski J. Diagnozowanie zjawiska przemocy domowej – konteksty teoretyczne i praktyczne. Family Forum. (2019) 3:1508 10.25167/FF/1508

[26] Sniegulska A. Violence against women and elderly people in a family environment. J Mod Sci. (2016) 4:101–24. Available online at: https://www.jomswsge.com/pdf-80062-16045?filename=Violence%20against%20women.pdf (accessed September 20, 2022).

[27] Miedzik M Godlewska-Szurkowska J Rutkowski J. Raport czastkowy. Zadanie nr 1 Badania porównawcze oraz diagnoza skali wystepowania przemocy w rodzinie wśród osób dorosłych i dzieci, z podziałem na poszczególne formy przemocy wraz z opisem charakterystyki ofiar przemocy i sprawców Wyd. Warszawa: Ministerstwo Pracy i Polityki Społecznej (2014).

[28] Ministerstwo Rodziny. Pracy i Polityki Społecznej. Ogólnopolska diagnoza infrastruktury wsparcia dla osób doznajacych przemocy oraz ocena efektywności i skuteczności stosowanych form pomocy. Warszawa: Raport końcowy (2017).

[29] von Elm E Altman DG Egger M Pocock SJ Gøtzsche PC Vandenbroucke JP. Strobe initiative the strengthening the reporting of observational studies in epidemiology (STROBE) statement: guidelines for reporting observational studies. J Clin Epidemiol. (2008) 61:344–9. 10.1016/j.jclinepi.2007.11.008 18313558

[30] Bows H. Practitioner Views on the Impacts, Challenges, and Barriers in Supporting Older Survivors of Sexual Violence. Violence Against Wom. (2018) 24:1070–90. 10.1177/1077801217732348 29332552 PMC6009174

[31] Tao X Ge SQ Chen L Cai LS Hwang MF Wang CL. Relationships between female infertility and female genital infections and pelvic inflammatory disease: a population-based nested controlled study. Clinics. (2018) 9:e364. 10.6061/clinics/2018/e364 30110069 PMC6077933

[32] Zarenezhad M Valee M Gholamzadeh S Malekpour A Keshavarz P. Severe and unusual domestic violence; a case report and review of literature. J Case Rep Stud. (2016) 4:1–3. 10.15744/2348-9820.4.601

[33] Ogińska-Bulik N. Objawy stresu pourazowego i potraumatyczny wzrost u kobiet doznajacych przemocy w rodzinie. Psychiatria i Psychoterapia. (2016) 12:15–29.

[34] Mirski A. Jakość zycia u kobiet i dzieci, które padły ofiara przemocy wrodzinie. Państwo i Społeczeństwo. (2015) 15:89–109.

[35] Karakuła-Juchnowicz H Łukasik P. Polska adaptacja i walidacja Skali Doświadczeń Maltretowanych Kobiet. Women's experience with battering scale-WEB. Scale Curr Probl Psychiatry. (2013) 14:201–5.

[36] Diener E Lucas R Oishi S. Subjective wellbeing: the science of happiness and life satisfaction. In C. R. Snyder and S. J. Lopez (Eds.), The Handbook of Positive Psychology. New York: Oxford University Press (2012) 63–73.

[37] Juczyński Z. Narzedzia pomiaru w promocji i psychologii zdrowia. Warszawa: Wyd Pracownia Testów Psychologicznych, Polskiego Towarzystwa Psychologicznego (2012).

[38] Pavot W Diener E. Review of the Satisfaction With Life Scale. [W:] Diener E. (red.): Assessing WellBeing: *The Collected Works of Ed Diener, Social Indicators Research Series 39*, Springer Science and Business Media B.V (2009) 101–117. 10.1007/978-90-481-2354-45

[39] Craparo G Gori A Petruccelli I Cannella V Simonelli C. Intimate partner violence: relationships between alexithymia, depression, attachment styles, and coping strategies of battered women. J Sex Med. (2014) 11:1484–94. 10.1111/jsm.12505 24621112

[40] Franczyk-Glita J Müldner-Nieckowski Ł Smierciak N Daren A Furgał M. Formy agresji kobiet doświadczajacych przemocy w relacji intymnej. Psychoterapia. (2018) 1:65–78.

[41] Cichla J. Dynamika i uwarunkowania przemian psychospołecznego funkcjonowania kobiet ofiar przemocy domowej w trakcie procesu terapeutycznego. Katowice: Rozprawa doktorska Uniwersytet Slaski, Wydział Pedagogiki i Psychologii. (2014).

[42] Sitarczyk M. Poczucie koherencji kobiet - ofiar przemocy domowej. Psychologia Jakości Zycia. (2013) 12:57–73. 10.5604/16441796.1088807

[43] Filipek A. Wspomaganie człowieka dorosłego w sytuacji przemocy w rodzinie. Wydział Pedagogiki i Psychologii. Białystok: Rozprawa doktorska Uniwersytet w Białymstoku (2017).

[44] Shaheen F. A Study of life satisfaction and optimism in relation to psychological well-being among working and non-working women. IJEPR. (2015) 4:81–5. Available online at: https://ijepr.org/panel/assets/papers/230ij18.pdf (accessed September 20, 2022).

[45] Banaei M Amir Ali Aliakbari S Ghalandari S Eslami K. Assess the comparison of marital satisfaction between the abused and non-abused women. Int J Med Res Health Sci. (2016) 5:617–24.

[46] Adams AE Beeble ML. Intimate partner violence and psychological well-being: Examining the effect of economic abuse on women's quality oflife. Psychol Viol. (2018) 3:174. 10.1037/vio0000174

[47] Alsaker K Moen BE Baste V. Intimate partner violence associated with low quality of life. BMC Women's Health. (2018) 17:147. 10.1186/s12905-018-0638-5 30180829 PMC6123959

[48] Gamal AM Hussein SR Ayoub GG. Quality of life among women who exposed to violence: adult women vs. old women. IOSR-JNHS. (2018) 7:73–84. 10.9790/1959-0706077384

[49] Gharacheh M Azadi S Mohammadi N Montazeri S Khalajinia Z. Domestic violence during pregnancy and women's health-related quality of life. Glob J HealthSci. (2016) 8:27–34. 10.5539/gjhs.v8n2p27 26383205 PMC4803975

[50] Ghasemi SR Reshadat S Rajabi-Gilan N Salimi Y. NorouziM.Therelationship between rural women's health-related quality of life and domestic violence. Zahedan J Res Med Sci. (2015) 17:e978. 10.17795/zjrms978

[51] Lucena KDT Vianna RPT Nascimento JA Coelho HFC Oliveira ECT. Association between domestic violence and women's quality of life. Rev Lat Am Enfermagem. (2017) 25:e2901. 10.1590/1518-8345.1535.2901 28591305 PMC5479378

